# The Approach to Steady State Using Homogeneous and Cartesian Coordinates

**DOI:** 10.1155/2013/729236

**Published:** 2013-07-28

**Authors:** D. F. Gochberg, Z. Ding

**Affiliations:** ^1^Department of Radiology and Radiological Sciences, Vanderbilt University Institute for Imaging Science (VUIIS), Vanderbilt University, Nashville, TN 37232-2675, USA; ^2^Department of Physics, Vanderbilt University Institute for Imaging Science (VUIIS), Vanderbilt University, Nashville, TN 37232-2675, USA

## Abstract

Repeating an arbitrary sequence of RF pulses and magnetic field gradients will eventually lead to a steady-state condition in any magnetic resonance system. While numerical methods can quantify this trajectory, analytic analysis provides significantly more insight and a means for faster calculation. Recently, an analytic analysis using homogeneous coordinates was published. The current work further develops this line of thought and compares the relative merits of using a homogeneous or a Cartesian coordinate system.

## 1. Introduction

A recent paper by Nazarova and Hemminga [1] analyzed the signal arising from repeated identical RF pulses using a formalism based on homogeneous coordinates. Unlike the conventional Cartesian coordinates, homogeneous coordinates allow *T*_1_ relaxation to be treated in a manner similar to RF field-induced rotations, that is, by matrix multiplication. Signals from repeated identical RF pulses are an issue, most notably, in steady-state free precession (SSFP) fast imaging [2] techniques, where the lack of gradient or RF spoiling leads to complicated dynamics when the repetition time <*T*_2_. The formalism can also be applied to any repeated sequence of pulses, such as those, for example, that arise in pulsed saturation magnetization transfer methods [3].

In this paper, we will expand on the work of Nazarova and Hemminga to show a simple way to calculate the steady-state magnetization, and we will relate the properties of the homogeneous matrices and calculations to those that arise in a more traditional Cartesian representation of magnetization. Specifically, we explicitly establish algebraic relations between the two systems, thus facilitating understanding of the pros and cons of each representation. We will employ the same notation as Nazarova and Hemminga, except as noted.

## 2. Background

**Figure 1:**
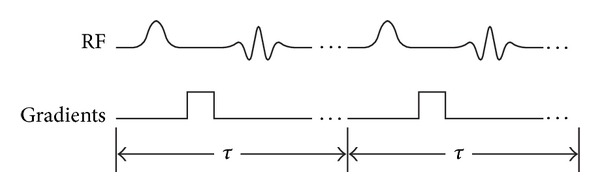
An arbitrary sequence of pulses repeated every *τ* seconds.

Consider a pulse sequence made up of RF pulses and magnetic field gradients repeated every *τ* seconds (see Figure 1). The conventional representation of the magnetization is a 3 × 1 vector **M** with equilibrium value **M**_eq_ and components *M*_*x*_, *M*_*y*_, and *M*_*z*_ in the rotating frame. RF pulses with field strengths = *B*_1_ ≫ 1/(*γT*_2_) and angular frequency offset Δ*ω*_0_ can be represented by a 3 × 3 rotation matrix *R*_*x*,*y*_(*α*), where *T*_2_ is the transverse relaxation rate and *γ* is the gyromagnetic ratio. Likewise, off-resonance precession and the scaling effect of relaxation can also be represented by matrices. For example, precession about the *z* direction and the decay of magnetization can be represented as follows:

(1) $$\begin{array}{c} R_{z}\left(\Delta \omega_{0}\tau \right)=\left[\begin{array}{ccc} \cos\Delta \omega_{0}\tau & \sin\Delta \omega_{0}\tau & 0\\ -\sin\Delta \omega_{0}\tau & \cos\Delta \omega_{0}\tau & 0\\ 0 & 0 & 1 \end{array}\right],\\ S\left(\tau ,T_{1},T_{2}\right)=\left[\begin{array}{ccc} e^{-\tau /T_{2}} & 0 & 0\\ 0 & e^{-\tau /T_{2}} & 0\\ 0 & 0 & e^{-\tau /T_{1}} \end{array}\right]. \end{array}$$

Similarly, a gradient pulse can be modeled by making the precession position dependent. In distinction, the effect of  *T*_1_ relaxation to a nonzero thermal equilibrium value cannot be represented by a 3 × 3 matrix. It is instead equivalent to an additive translation.

Nazarova and Hemminga use this approach to give the relation for magnetization after *n* repeated *α* pulses about the *x*-axis:

(2) $$\begin{array}{c} M_{n}=R_{z}\left(\Delta \omega_{0}\tau \right)S\left(\tau ,T_{1},T_{2}\right)R_{x}\left(\alpha \right)M_{n-1}+\left(1-e^{-\tau /T_{1}}\right)M_{\text{eq}}, \end{array}$$

where **M**_*n*_ is the magnetization after the *n*th repetition (initial condition = **M**_0_). The general form for the repetition of an arbitrary pulse sequence is

(3) $$\begin{array}{c} M_{n}=CM_{n-1}+DM_{\text{eq}},\;\text{for}\;\;n\geq 1,\\ M_{n}=M_{0},\;\text{for}\;\;n=0. \end{array}$$

Note that **D** is scalar only when a single RF pulse is repeated.

An alternative approach is to use homogeneous coordinates where *T*_1_ relaxation enters in the same way as do rotations:

(4) $$\begin{array}{c} M_{n}=AM_{n-1},\;\text{for}\;\;n\geq 1,\\ M_{n}=M_{0},\;\text{for}\;\;n=0. \end{array}$$

**M** is now a 4 × 1 vector with components *M*_*x*_, *M*_*y*_, *M*_*z*_, and 1, and **A** is a 4 × 4 matrix whose upper left portion matches the 3 × 3 rotations and scalings discussed above and whose far right column represents the *T*_1_ relaxation towards **M**_eq_. For example,

(5) $$\begin{array}{c} R_{z}\left(\Delta \omega_{0}\tau \right)=\left[\begin{array}{cccc} \cos\Delta \omega_{0}\tau & \sin\Delta \omega_{0}\tau & 0 & 0\\ -\sin\Delta \omega_{0}\tau & \cos\Delta \omega_{0}\tau & 0 & 0\\ 0 & 0 & 1 & 0\\ 0 & 0 & 0 & 1 \end{array}\right]. \end{array}$$

And *T*_1_ relaxation for a period *τ* is represented by

(6) $$\begin{array}{c} T=\left[\begin{array}{cccc} 1 & 0 & 0 & 0\\ 0 & 1 & 0 & 0\\ 0 & 0 & 1 & M_{\text{eq}}\left(1-e^{-\tau /T_{1}}\right)\\ 0 & 0 & 0 & 1 \end{array}\right]. \end{array}$$

**M**_eq_ in this notation is represented by

(7) $$\begin{array}{c} \left[\begin{array}{c} 0\\ 0\\ M_{\text{eq}}\\ 1 \end{array}\right]. \end{array}$$

(Note the corrections to Nazarova and Hemminga in (6) and (7)).

## 3. Homogeneous and Cartesian Representations

Both homogeneous and conventional representations have identical underlying math and therefore will give equivalent results. However, their differences in formalism have small consequences in computation time, and they provide different notational approaches.

The solution in the homogeneous case is

(8) Mn=AnM0=pA1λA1nvA1+pA2λA2nvA2+pA3λA3nvA3+pA4λA4nvA4,

where **v**_*Ai*_ and *λ*_*Ai*_ are the *i*th eigenvector and eigenvalue, respectively, of **A** and *p*_*Ai*_ is the corresponding projection of **M**_0_ onto **v**_*Ai*_. The second form of the solution can be derived in two ways: (1) by rewriting **A** ^*n*^ as (**V**_*A*_Λ_*A*_ **V**_*A*_^−1^)^*n*^ = **V**_*A*_Λ_*A*_^*n*^ **V**_*A*_^−1^, where the columns of **V**_*A*_ are **v**_*Ai*_, Λ_*A*_ is diagonal with elements *λ*_*Ai*_, and **p**_*A*_ (with elements *p*_*Ai*_) equals **V**_*A*_^−1^ **M**_0_ and (2) by rewriting **M**_0_ as *p*_*A*1_ **v**_*A*1_ + *p*_*A*2_ **v**_*A*2_ + *p*_*A*3_ **v**_*A*3_ + *p*_*A*4_ **v**_*A*4_ and applying (4) *n* times. (Note that in Nazarova and Hemminga **V**_*A*_ is called **B**).

The conventional Cartesian case has the less intuitive solution to (3):

(9) MnCnM0+(∑i=0n−1Ci)DMeq, for  n≥1=CnM0+(−∑i=n∞CiD+∑i=0∞CiD)Meq=CnM0+(−Cn+I)∑i=0∞CiDMeq=Cn(M0−(I−C)−1DMeq)+(I−C)−1DMeq

(10) VCΛnVC−1(M0−(I−C)−1DMeq)+(I−C)−1DMeq=pC1λC1nvC1+pC2λC2nvC2+pC3λC3nvC3+(I−C)−1DMeq,

where the columns of **V**_*C*_ are the eigenvectors **v**_*Ci*_ of **C**, the elements of the diagonal matrix Λ_*C*_ are the eigenvalues *λ*_*Ci*_, and **p**_*C*_ (with elements *p*_*Ci*_) equals **V**_*C*_^−1^(**M**_0_ − (**I**−**C**)^−1^ **D** **M**_eq_).

Often, one only cares about the steady-state solution, which is the solution that is independent of *n* and is approached as *n* → *∞*. We can solve this condition by solving **M**_*n*_ when **M**_*n*_ = **M**_*n*−1_. For the homogeneous notation (4),

(11) $$\begin{array}{c} M_{n}=AM_{n-1}=AM_{n}. \end{array}$$

That is, the steady-state solution is the eigenvector of **A** with eigenvalue = 1. 

In the conventional formalism, we again solve for a steady-state solution (≡**M**_ss_) by setting **M**_*n*_ = **M**_*n*−1_, this time in (3):

(12) $$\begin{array}{c} M_{\text{ss}}={\left(I-C\right)}^{-1}DM_{\text{eq}}, \end{array}$$

(another option is to take *n* → *∞* in (9) with **C** ^*n*^ → 0 due to the relaxation terms on the diagonal). Since taking an inverse takes ~dimension^3^ operations, while finding an eigenvector via row reduction takes ~1/3 dimension^3^ operations [4], finding the steady-state via the homogeneous equation may provide a slightly more efficient method, though no rigorous evaluation of the computation time has been made. The computation time is normally not essential but may be so in certain problems, such as least squares fitting of magnetization transfer parameters [3].

## 4. Relations between Homogeneous and Cartesian Representations

In general, **A** has the form

(13) $$\begin{array}{c} A=\left[\begin{array}{cccc} & C\left(3\times 3\right) & & DM_{\text{eq}}\left(3\times 1\right)\\ 0 & 0 & 0 & 1 \end{array}\right]. \end{array}$$

From this form it follows that

(14) $$\begin{array}{c} \lambda_{Ai}=\lambda_{Ci},\;\text{for}\;\;i=1,2,3,\\ \lambda_{A4}=1,\\ v_{Ai}=\left[\begin{array}{c} v_{Ci}\\ 0 \end{array}\right],\;\text{for}\;\;i=1,2,3,\\ v_{A4}=\left[\begin{array}{c} M_{\text{ss}}\\ 1 \end{array}\right]. \end{array}$$

## 5. An Example

Consider a simple example: a single short (relative to *T*_2_) 90° pulse repeated every *τ* seconds. To keep the illustration as analytically simple as possible, we assume *T*_1_ = *T*_2_ and a system starting at thermal equilibrium. This gives (from Nazarova and Hemminga with corrections)

(15) $$\begin{array}{c} A=\left[\begin{array}{cccc} E\cos\theta & 0 & E\sin\theta & 0\\ -E\sin\theta & 0 & E\cos\theta & 0\\ 0 & -E & 0 & M_{\text{eq}}\left(1-E\right)\\ 0 & 0 & 0 & 0 \end{array}\right], \end{array}$$

where *E* = exp⁡(−*τ*/*T*_1_) = exp⁡(−*τ*/*T*_2_) and *θ* = Δ*ω*_0_ *τ*. We can solve **M**_*n*_ using (8) and then convert back to Cartesian coordinates, or we can use (13) to get **C** and **D** **M**_0_ and then use (10). With help from Mathematica, we get

(16) Mn=Meq1+E2+2Eγ[2Eγ(1−γ)E(1−E−2γ)1+E(−1+2γ)]+(−E)n(γ+i1−γ2)n×Ei1−γ2(i1−γ2+E+γ)Meq(1+γ)(1+E2+2Eγ)(−1+γ+i1−γ2)×[2γ(1−γ)−1+γ−i1−γ2γ+i1−γ21]+En(−γ+i1−γ2)n×Ei1−γ2(−i1−γ2+E+γ)Meq(1+γ)(1+E2+2Eγ)(1−γ+i1−γ2)×[2γ(1−γ)−1+γ+i1−γ2γ−i1−γ21],

where *γ* = (1 − cos⁡*θ*)/2 (not to be confused with the gyromagnetic ratio). Note that even though it is calculated from complex eigenvectors and eigenvalues, for every *n*, **M**_*n*_ has real components. (We have chosen to keep the solution in a form where its connection to (8) is clear.) One of the eigenvalues = 1, corresponding to the steady-state solution. Since one of the *p* components equals zero, there are only two eigenvectors that contribute, thereby ensuring that **M**_*n*_ travels in a plane as n increases. If there is no relaxation (*E* = 1), then this plane is perpendicular to the steady-state vector, as can be seen by taking the dot product of the steady-state vector with the other two eigenvectors. (In this case the “steady-state” vector is never actually reached, as discussed below.) 

If instead **M**_0_ ≠ **M**_eq_, then none of the *p* components are equal to zero. Therefore, an additional eigenvector contributes, and the path is no longer planar. The additional eigenvector is

(17) $$\begin{array}{c} \left[\begin{array}{c} \sqrt{\frac{\gamma -1}{\gamma }}\\ -1\\ 1 \end{array}\right] \end{array}$$

with corresponding eigenvalue *E*. Since this eigenvalue is real, it cannot contribute to a rotational trajectory, as will be illustrated below.

## 6. Numerical Methods

**Figure 2:**
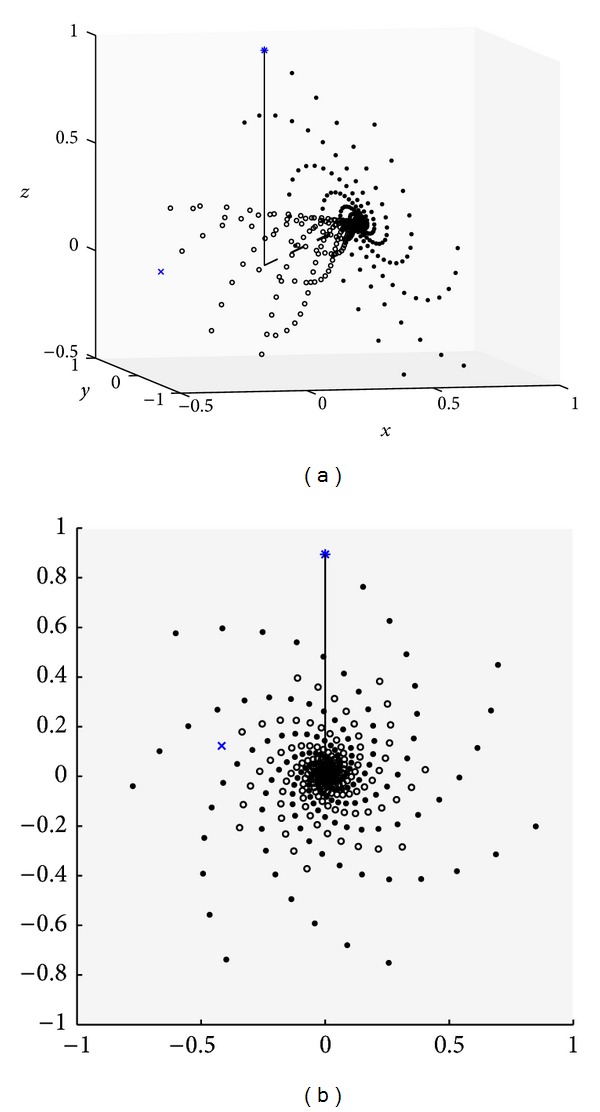
**M**_*n*_ for *n* = 0,1,…, 200. *α* = 90°, *θ* = 60°, and *E* = 0.98 with two different initial conditions: **M**_0_ = [0 0 1] = **M**_eq_ (solid circles starting at ∗) and [−0.4472 −0.2236 0] (open circles starting at ×). The solid line is along the +*z*-axis and the dashed line is the steady-state magnetization **M**_ss_. (a) and (b) are two different views, with (b) looking down along the direction of **M**_ss_.

**Figure 3:**
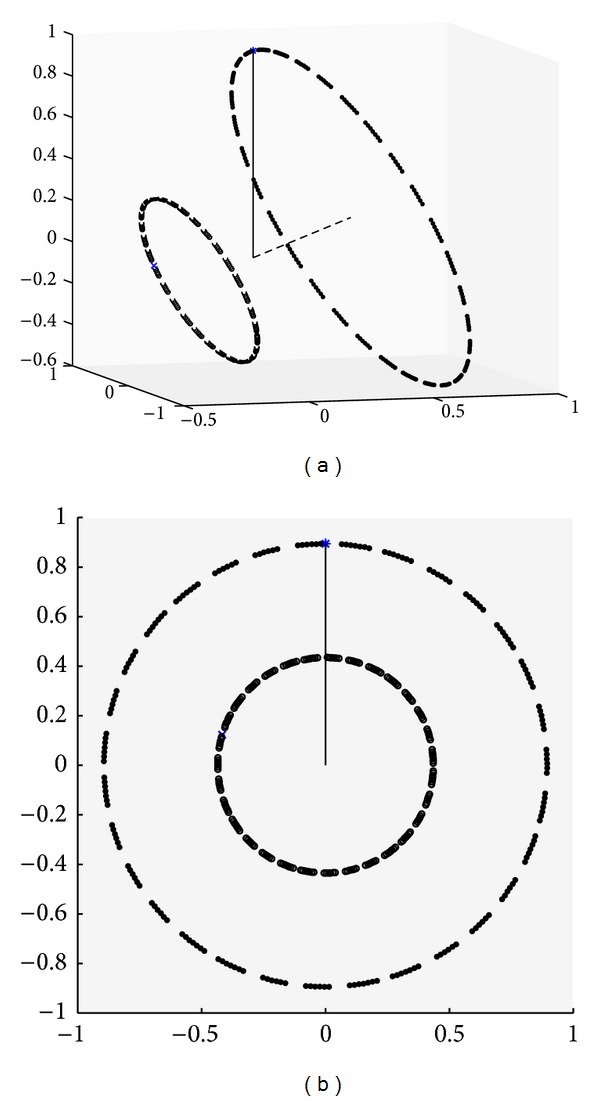
Identical to Figure 2, but with *E* = 1. Note that the “steady-state” vector is never actually reached and that it is perpendicular to the plane containing the circular trajectory.

Figure 2 plots the case for a repeated 90° pulse with *E* = 0.98, *θ* = 60°, and with two different initial conditions **M**_0_.  Figure 2(a) illustrates that when **M**_0_ = **M**_eq_, the path is a planar spiral, as expected. When **M**_0_ ≠ **M**_eq_ (reached by magnetization preparations), the spiral instead wraps around a cone. The head-on view in Figure 2(b) indicates that the spirals rotate at the same angular rate, which follows from the additional eigenvalue being real. The conditions in Figure 3 are the same as in Figure 2 except that *E* = 1. With no relaxation, there is no decay and the “steady-state” solution is never reached; it is perpendicular to the plane containing the trajectory.

## 7. Discussion

The homogeneous and Cartesian coordinates provide two equivalent ways of representing magnetization. In pulsed repetition experiments, homogeneous coordinates lead to a simpler equation for the dynamics and steady state, but with a less intuitive connection to the measured magnetization. The choice of which system to use is in the end one of personal preference.
